# An enhanced adaptive dynamic metaheuristic optimization algorithm for rainfall prediction depends on long short-term memory

**DOI:** 10.1371/journal.pone.0317554

**Published:** 2025-06-02

**Authors:** Ahmed M. Elshewey, Amel Ali Alhussan, Doaa Sami Khafaga, Marwa Radwan, El-Sayed M. El-kenawy, Nima Khodadadi

**Affiliations:** 1 Department of Computer Science, Faculty of Computers and Information, Suez University, Suez, Egypt; 2 Department of Computer Sciences, College of Computer and Information Sciences, Princess Nourah bint Abdulrahman University, Riyadh, Saudi Arabia; 3 Faculty of Artiﬁcial Intelligence, Delta University for Science and Technology, Mansoura, Egypt; 4 School of ICT, Faculty of Engineering, Design and Information & Communications Technology (EDICT), Bahrain Polytechnic, Isa Town, Bahrain; 5 Applied Science Research Center. Applied Science Private University, Amman, Jordan; 6 Department of Civil and Architectural Engineering, University of Miami, Coral Gables, Florida , United States of America; Torrens University Australia, AUSTRALIA

## Abstract

Sorting and analyzing different types of rainfall according to their intensity, duration, distribution, and associated meteorological circumstances is the process of rainfall prediction. Understanding rainfall patterns and predictions is crucial for various applications, such as climate studies, weather forecasting, agriculture, and water resource management. Making educated decisions about things like agricultural planning, effective use of water resources, precise weather forecasting, and a greater comprehension of climate-related phenomena is made more accessible when many components of rainfall are analyzed. The capacity to confront and overcome this obstacle is where machine learning and metaheuristic algorithms shine. This study introduces the Adaptive Dynamic Particle Swarm Optimization enhanced with the Guided Whale Optimization Algorithm (AD-PSO-Guided WOA) for rainfall prediction. The AD-PSO-Guided WOA overcomes limitations of conventional optimization algorithms, such as premature convergence by balancing global search (exploration) and local refinement (exploitation). This effectively balances exploration and exploitation, and addresses the early convergence problem of the original algorithms. To choose the most crucial characteristics of the dataset, the feature selection method employs the binary format of AD-PSO-Guided WOA. Next, the desired features are trained on five different models: Decision Trees (DT), Random Forest (RF), Multi-Layer Perceptron (MLP), Long Short-Term Memory (LSTM), and K-Nearest Neighbor (KNN). Out of all the models, LSTM produced the best results. The AD-PSO-Guided WOA algorithm was used to adjust the hyperparameters for the LSTM model. With coefficient of determination (R^2^) of 0.9636, the results demonstrate the superior efficacy and performance of the suggested methodology (AD-PSO-Guided WOA-LSTM) compared to other alternative optimization techniques.

## Introduction

Predicting rainfall is a crucial and challenging task in the field of meteorological forecasting, with implications for many different industries, including agriculture, water resource management, and disaster preparedness [1]. Making well-informed decisions to minimize risks and maximize resource use requires accurate and timely rainfall pattern forecasts [2]. Accurately predicting rainfall patterns is crucial for well-informed decision-making that affects the public’s safety and the economy [3]. Accurate rainfall prediction is crucial for many research projects because fast and reliable forecasting of extreme weather can be crucial for reducing fatalities and property damage from natural catastrophes. Researchers in various fields, such as operational hydrology, environmental machine learning, meteorological data mining, and statistical forecasting, have a great problem in creating a predictive system for precise rainfall forecasting. Building such a system is essential to improving our capacity to issue early warnings, which in turn helps with better preparedness and mitigation efforts for disasters [4]. Rainfall forecasting is a challenging and uncertain process that has a significant influence on human culture. Proactively reducing financial and human losses requires precise and timely forecasts. Since the Meteorological Bureau is closely related to the economy and people’s livelihoods, it faces many obstacles, especially when forecasting severe rains. This component of meteorological forecasting is directly related to the annual recurrence of natural disasters, such as droughts and floods, that affect the entire world. As global warming continues, detecting and predicting rainfall is becoming increasingly important, especially in areas without access to adequate equipment [5]. When accurate forecasts are made, they can accomplish several goals, including promoting public health, assisting with access to drinking water, and assisting with agriculture. Precipitation forecast accuracy becomes critical, particularly in nations primarily relying on agriculture. Traditional statistical methods sometimes fail to provide high accuracy for precipitation predictions due to the dynamic nature of the environment. Because precipitation data is non-linear, machine learning (ML), artificial intelligence (AI), and metaheuristic optimization algorithms are more practical methods. The significance of precision in meteorological forecasts is highlighted by the necessity of accurate predictions, which allow people to take preventative steps [6]. Rainfall prediction technology that is accurate and non-invasive is desperately needed. Intricate and nonlinear linkages in rainfall circumstances have been revealed by machine learning (ML), deep learning (DL), and metaheuristic optimization algorithms, all of which have shown effectiveness in the process of rainfall prediction [3]. A critical step in building a machine-learning model is hyperparameter tuning, choosing the best values for parameters that affect how the algorithm behaves during training. The model’s learning rate, regularization, number of layers, and nodes within each layer are all controlled by these hyperparameters [7]. The accuracy, generalization, and convergence speed of the model are all significantly impacted by the proper selection of hyperparameters. Prudent hyperparameter selection improves the model’s ability to generalize and make precise predictions about unknown inputs. On the other hand, incorrect hyperparameters might lead to underfitting, overfitting, or poor performance. Hyperparameter optimization is crucial to improve the machine learning model’s performance and make it more reliable, precise, and efficient [8]. The application of LSTM in rainfall prediction has shown it to be a potent technique. Rainfall pattern prediction is especially well-suited for this particular kind of recurrent neural network (RNN), as it has demonstrated efficacy in capturing and comprehending temporal correlations within time series data. By addressing the problem of vanishing gradients that conventional RNNs encounter, LSTM makes it possible to remember and apply information over longer sequences [9]. Regarding rainfall forecasting, LSTM models demonstrate the capacity to identify intricate correlations in past rainfall data, resulting in more precise forecasts. Rainfall patterns can exhibit both short-term variations and long-term trends, which can be captured by LSTMs through processing and learning from previous data. Their proficiency in managing the dynamic and non-linear characteristics of meteorological data stems from this [10]. Researchers frequently use LSTM architectures to anticipate rainfall at different temporal scales, from longer-term projections to shorter-term forecasts [11]. LSTMs are useful for comprehending the dynamic nature of weather patterns because they can capture sequential dependencies in data, eventually improving the accuracy of rainfall predictions [12]. LSTM models are trained on historical rainfall data in practical applications, taking wind speed, temperature, and humidity into account. The LSTM can be used to forecast future time points after it has been trained. Our capacity to anticipate and prepare for weather-related occurrences has improved thanks to the versatility and efficiency of LSTM in managing the intricacies of rainfall prediction [13].

This paper presents an improved method that relies on LSTM to predict rainfall in Australia. Specifically, we use the AD-PSO-Guided WOA-LSTM algorithm to predict rainfall while taking into account a wide range of features.

### Related work

Endalie et al. [14] introduced an artificial neural network was employed to construct a rainfall forecasting model specifically for South Korea’s Geum River Basin during the late spring/early summer period. The optimal artificial neural network model demonstrated relative root mean square errors of 25.84 percent, 32.72 percent, and 34.75 percent for the training, validation, and testing datasets, respectively. This suggests the accurate prediction of rainfall in the study region, as indicated by a hit score exceeding 60%, calculated as the number of hit years divided by the total number of years. El-Shafie et al. [15] demonstrated an artificial neural network for forecasting the rainfall-runoff dynamics within a catchment area situated in the Tanakami region of Japan. The research highlights the utilization of a feedforward backpropagation architecture, employing hyperbolic tangent neurons in the hidden layer and linear neurons in the output layer for rainfall prediction. To assess the model’s efficacy, three statistical metrics, namely the correlation coefficient, mean square error, and correlation of determination, were utilized. The outcomes indicate that the feedforward backpropagation neural network can accurately depict the behavior of the rainfall-runoff relationship, surpassing the performance of the conventional regression model with R^2^ value equals 0.99. Hasan et al. [16] presented a robust approach for rainfall prediction using support vector regression based on recent rainfall data from Bangladesh. support vector regression is a regression methodology derived from support vector machine. The raw data collected underwent manual preprocessing to align with the algorithm’s input requirements before being fed into the algorithm. Evaluation results from the study demonstrate that the proposed technique outperforms traditional frameworks in terms of accuracy and processing time. The suggested approach achieved a maximum prediction of 0.992. He et al. [17] introduced a rainfall forecasting model that incorporates multi-resolution analysis and multiple linear regression using monthly historical rainfall data and climate indices. The findings reveal that the proposed multi-resolution analysis based model consistently provides more accurate monthly rainfall forecasts for all selected stations in South Australia compared to the traditional regression model. Historical rainfall data proves consistently useful across all stations in the multi-resolution analysis -based method, while large-scale climate signals are only partially beneficial for certain stations. Ramana [18] proposed a study over a 10-year period in the study area, the average runoff has been computed as 54.74% and 51.50% of rainfall using the soil conservation service curve number method and the technical release 55 model, respectively. This suggests a 3.31% overestimation in the average yearly runoff by the soil conservation service curve number method in comparison to the technical release 55 model. The integration of geographic information system with the technical release 55 model enhanced the accuracy and efficiency of runoff estimation. Consequently, the runoff estimated using the technical release 55 model was determined to be in close agreement with the observed runoff. Similarly, the runoff estimation derived from the geographic information system and remote sensing-based soil conservation service curve number method aligned well with observed runoff, offering valuable support for improved water management practices. Hong et al. [19] introduced an innovative forecasting approach namely, support vector regressor simulated annealing algorithm, designed for predicting rainfall levels during typhoon seasons in northern Taiwan. Experimental findings indicate the support vector regressor simulated annealing algorithm as a promising and effective option for rainfall forecasting. The superior performance of the support vector regressor simulated annealing algorithm can be attributed to several factors. Firstly, support vector regressor emphasizes structural risk minimization over solely minimizing training errors, contributing to robust generalization capabilities. Secondly, the use of simulated annealing algorithm facilitates the proper selection of three parameters in the support vector regressor simulated annealing algorithm, thereby enhancing forecasting accuracy. This study underscores the validity of the proposed support vector regressor simulated annealing algorithm as a valuable model. Goyal [20] presented a wavelet regression technique for the analysis and prediction of rainfall forecasts. The enhanced wavelet regression model combines two methodologies: discrete wavelet transforms and linear regression. Rainfall data from 21 stations in Assam, India, spanning 102 years (1901–2002), is employed for this study. The models undergo calibration and validation, and their performance is assessed using appropriate statistical methods. Evaluation metrics such as root mean square errors and correlation coefficient are utilized to gauge the accuracy of the wavelet regression models. Furthermore, the accuracy of the wavelet regression models is compared with that of artificial neural networks models. The outcomes from the modeling of monthly rainfall series reveal that the wavelet regression models exhibit greater accuracy compared to the artificial neural networks models. Danandeh et al. [21] proposed the fusion of support vector regression and firefly algorithm to generate accurate and reliable rainfall predictions. The hybrid model was trained and validated using the weak stationary state of monthly rainfall data obtained from various gauges. To assess its efficiency, the model’s results were cross-validated against stand-alone support vector regression and genetic programming-based forecasting models, which served as benchmarks in this study. Results for both rain gauge locations demonstrated the significant superiority of the hybrid model over the benchmarks. In terms of average efficiency results at the gauge locations, the firefly algorithm -induced enhancement in support vector regression forecasts corresponded to an approximately 30% reduction in root-mean-square error and an approximately 100% increase in Nash–Sutcliffe efficiency. Pai et al. [22] proposed the application of support vector regression models and recurrent support vector regression models has proven successful in addressing time-series challenges in various domains. Despite this, the utilization of recurrent support vector regression models in rainfall forecasting remains relatively unexplored. This study aims to enhance the accuracy of rainfall forecasting by leveraging the unique capabilities of the support vector regression model, genetic algorithms, and the recurrent network architecture. The study explores the performance of genetic algorithms with varying mutation rates and crossover rates in the selection of support vector regression parameters. Simulation results highlight the recurrent support vector regression with genetic algorithms model as an effective approach for predicting rainfall amounts. Hossain et al. [23] developed non-linear models involved utilizing past values of climate drivers that exhibit a significant correlation with rainfall. Specifically, the effectiveness of South-eastern Indian Ocean and El Niño Southern Oscillation in replicating rainfall characteristics was assessed through a non-linear regression approach. These models underwent testing using individual datasets not utilized during the calibration phase. Evaluation was conducted using standard statistical parameters, including pearson correlations, root mean square error, mean absolute error, and index of agreement. A case study focused on three rainfall stations in the australian capital territory. The analysis revealed that predictors with the highest correlation with predictands do not necessarily result in the least errors in rainfall forecasting. The non-linear regression successfully predicted seasonal rainfall, with correlation coefficients ranging from 0.71 to 0.91. Chandniha et al. [24] emphasized the application of the multiple linear regression-based statistical downscaling model technique for evaluating future monthly rainfall in the Piperiya watershed located in Chhattisgarh state, India. The tool is commonly employed for hydro-meteorological downscaling of global climate models to local fine-scale resolutions. In this study, daily rainfall time series corresponding to Hadley Centre Coupled Model version 3 emission scenarios are generated and utilized for estimating monthly rainfall in various future time periods. Model calibration and validation are conducted using NCEP reanalysis data for the periods 1961–1990 and 1991–2001, respectively. The anticipation is that this research will contribute to effective water resource management in the state overall, particularly in the Piperiya watershed. Additionally, it aims to facilitate the examination of climate change effects on expected rainfall in this specific area.

In comparison to the related work, the AD-PSO-Guided WOA-LSTM algorithm that integrates more advanced hybrid optimization techniques that provide better computational efficiency and more prediction accuracy. Our proposed model addresses the shortcomings found in previous studies such as overfitting, early convergence, and total computational cost through employing a dynamic and adaptive approach that improves both accuracy and scalability. The previous studies rely on traditional regression and wavelet-based models and our proposed model leverages the temporal modeling strength of LSTM combined with feature optimization to achieve superior R^2^ of 0.9636.

## Materials and methods

### Dataset

The dataset used in this paper is available at [25]. The dataset encompasses a decade’s worth of daily weather data collected from numerous weather stations across Australia. It consists of 23 features and 145460 instances. The features are date, location, minimum temperature, maximum temperature, rainfall, evaporation, sunshine, wind gust direction, wind gust speed, wind direction at 9 am, wind direction at 3 pm, wind speed at 9 am, wind speed at 3 pm, relative humidity at 9 am, relative humidity at 3 pm, atmospheric pressure reduced to mean sea level at 9 am, atmospheric pressure reduced to mean sea level at 3 pm, fraction of sky obscured by cloud at 9 am, fraction of sky obscured by cloud at 3 pm, temperature at 9 am, temperature at 3 pm, rain today, and rain tomorrow. This dataset, which includes daily weather data from many weather stations throughout Australia spanning ten years, is essential for rainfall forecasting.

### Data preprocessing

Data preprocessing, which includes cleaning, converting, and integrating data to get it ready for analysis, is essential to the machine learning and metaheuristics processes [26]. To improve raw data suitability for upcoming analytical tasks, especially in machine learning and metaheuristic applications data preprocessing entails several crucial steps that refine transformation and integrate the data [26]. This phase’s main goal is to improve data quality and customize it to the needs of the intended data mining or modeling project. Preprocessing once thought of as a crucial basis for efficient data mining has changed to meet the growing complexity and requirements of contemporary machine learning, AI, and optimization algorithms [27]. Significant inconsistencies and irregularities are commonly found in real-world datasets which are frequently the result of various data generation manipulation and storage sources [28]. Missing values typos in manual entries redundant or duplicate records and inconsistently labeled entities representing the same concept are some examples of these inconsistencies. Because unprocessed data frequently lacks the structure or dependability required for reliable machine learning and metaheuristic optimization these problems present significant challenges. Advanced preprocessing pipelines use automated techniques to detect mitigate and correct these anomalies in a systematic manner to address these challenges. Although simple discrepancies can occasionally be resolved by hand algorithmic solutions that can preprocess data accurately and efficiently are required due to the size and complexity of contemporary datasets. Encoding for categorical variables scaling and normalization to standardize feature ranges imputation for missing values and algorithms to detect and manage outliers are all examples of automated workflows. To improve generalization and predictive accuracy these procedures not only improve data integrity but also maximize its compatibility with downstream learning algorithms [29]. Preprocessing continues to be a crucial component of effective machine learning and optimization pipelines because it bridges the gap between unstructured raw data and the exacting requirements of computational modeling. Its development keeps up with the increasing complexity and adaptability of modern analytical methods.

### Individual models

#### Long short-term memory.

Long Short-Term Memory (LSTM), which is a form of RNN, uses sequential information in their training, which permits data to pass from input to output neurons in the network [30]. Loops are added to the hidden layer of this design so that data can go both forward and backward, representing previous data at particular time steps. However, long-term dependencies are complex for RNNs to handle, mainly because of diminishing gradients, which make it difficult for the network to gather data from far-off phases. As the gradient gets closer to zero, adding more layers with activation functions worsens this problem [31]. This restriction is overcome by LSTM neural networks (LSTM-ANNs), which incorporate a memory unit and gate mechanism. With the help of this innovation, the network can now selectively store or discard data, making it easier to identify long-term dependencies in a series. LSTM networks show selective memory retention and forgetting using structures like cell states and three gates [32]. They excel at managing sequences with long temporal dependencies because of their special architecture, which enables them to learn dependencies over thousands of time steps. When selecting the hyperparameters of LSTM, quite a number of important parameters can significantly affect the model’s performance in solving a particular problem. The above hyperparameters are depicted in Table 1 below.

**Table 1:** Hyperparameters of the LSTM Model.

Hyperparameter	Description	Boundary settings
**Number of nodes**	Number of neurons or hidden units in each LSTM layer	128
**Number of hidden layers**	Number of layers between the input and output layers	4
**Number of units in dense layer**	Number of neurons in the dense layer	256
**Dropout**	Fraction of neurons to randomly drop out during training to prevent overfitting	0.2
**Weight initialization**	Method for initializing the weights of the network	Uniform
**Decay rate**	Rate at which weights decay to zero exponentially to prevent them from growing too large	1e^-6^
**Activation function**	Function that defines the output of a node as ON or OFF	Relu
**Learning rate**	Rate at which the network updates its parameters during training	1e^-5^
**Momentum**	Hyperparameter that allows accumulation of gradients from past steps to guide the search direction	0.7
**Number of epochs**	Number of complete iterations of the dataset during training	100
**Batch size**	Number of samples processed before updating internal parameters of the model	32

#### Multi-layer perceptron.

An artificial neural network with numerous layers of connected nodes is called a Multi-Layer Perceptron (MLP) model [33]. Its architecture consists of an input layer that receives input characteristics as its first step, one or more hidden layers that act as middle layers, processing data and linking nodes between layers, and an output layer that produces the network’s output. In contrast to simpler models, MLPs can identify complex patterns and correlations in data, which makes them useful in a variety of applications. Neurons in the network function as computational nodes, applying weights and biases to inputs. The model becomes more complex due to the activation function, which is usually non-linear, like a hyperbolic tangent or sigmoid. Weights that are changed during training are connected between nodes, and every node has a bias term that adds to the flexibility of the model [34]. Data moves through the network during training, with weights and biases applied to each layer to process inputs. Comparing the desired output of the network with its output results in error computation. Errors are propagated backward, and weights are adjusted to reduce the errors. Through supervised learning, MLP learns to recognize patterns by fine-tuning weights and biases in response to observed errors. MLP is widely used in machine learning due to its adaptability, which allows it to be applied to various problems [35].

#### K-nearest neighbor.

A flexible model that may be used for both regression and classification applications is K-Nearest Neighbors (KNN). It is frequently referred to as KNN regression in the context of regression [36]. By computing the average or weighted average of the target values among its K-nearest neighbors, the KNN regression model forecasts the numerical value of a target variable for a given data point. Measuring the distance between each data point in the training set and the one being considered for prediction is the first step in the procedure. Manhattan distance and Euclidean distance are frequently used distance measures [37]. The average, or weighted average, of the target values of the K-nearest neighbors is usually used to predict the value of the target variable in regression scenarios. Because smaller values tend to make the model more sensitive to individual data points, choosing a suitable value for K is crucial [37].

#### Decision tree.

One popular and adaptable supervised machine-learning technique used for regression and classification tasks is the Decision Tree (DT) model [38]. DT uses a tree-like hierarchical structure. Leaf nodes show the final predicted value, branches indicate outcomes, and each internal node acts as a decision point based on a feature. The algorithm evaluates a feature at each decision node and decides based on parameters such as information gain or Gini impurity. Until a predetermined halting condition is satisfied, this procedure iterates. Selecting criteria for node splitting is crucial; typical metrics are Gini impurity for regression tasks and mean squared error for mean squared errors. The goal is to maximize node homogeneity [39]. DT offers a measure of feature relevance, emphasizing traits that drastically lower variance or impurity as having greater decision-making power. DT is pruned, which involves removing branches that offer little predictive value to resolve overfitting and capture noise in training data and promote a more generic model [40]. The interpretability of DT is one of its main advantages. The produced tree structure provides a clear picture of the decision-making process. DT uses several splitting algorithms for different types of data, demonstrating flexibility in handling both numerical and categorical data [40].

#### Random forest.

A machine-learning model called the Random Forest (RF) regressor is intended for regression tasks [41]. It is categorized as ensemble learning and works by building a large number of decision trees in the training stage. The average forecasts of these different trees—predictions that are specifically designed for regression problems that provide the basis for the RF model’s final prediction. During training, RF, being an ensemble model, generates a variety of decision trees. To reduce the possibility of overfitting, each tree is constructed using a subset of the training data and randomly chosen features [42]. The bagging method used by the RF model involves creating several bootstrap samples (random subsets with replacement) from the training dataset. A different bootstrap sample is then used to train each decision tree. Furthermore, a random subset of features is taken into account for splitting at each decision tree node [43]. By purposefully adding unpredictability, the trees’ correlations are lessened, improving the ensemble’s performance.

### Proposed AD-PSO-guided WOA algorithm

This section delves into the Adaptive Dynamic Particle Swarm Optimization enhanced with the Guided Whale Optimization Algorithm (AD-PSO-Guided WOA) , employs an adaptive dynamic technique, the particle swarm algorithm, and a modified whale optimization algorithm. Algorithm 1 provides a depiction of the AD-PSO-Guided WOA algorithm.

#### Adaptive dynamic algorithm.

After the optimization method is initialized, each solution in the population is given a fitness value. The algorithm determines the optimal agent to be the solution with the highest fitness value. The first step in the adaptive dynamic process is to divide the population into two groups. The terms “exploration group” and “exploitation group” relate to these two groups. While members of the exploration group seek to investigate the area surrounding the leaders, members of the exploitation group have the primary goal of moving toward the optimal or best answer. The agents in the population groups dynamically update each other. To preserve an equilibrium between the exploration and exploitation groups, the optimization process starts with a 50/50 starting population distribution [44].

#### Guided WOA algorithm.

The WOA algorithm’s effectiveness is shown in various optimization tasks. It is recognized as one of the most potent optimization algorithms in the literature [44]. However, its restricted capacity for exploration could be a disadvantage [45,46]. Let *n* stand for the dimension, or the number of variables, in the search space that whales navigate for mathematical reasons. The ideal solution will be found if we take into account that the positions of agents or solutions in the search space change with time. Equation (1) is used to update the positions of agents in the WOA algorithm:


$$\vec{X}(t+1)={\vec{X}}^{*}(t)-\vec{A}.\vec{D},\vec{D}=\left|\vec{C}.{\vec{X}}^{*}(t)-\vec{X}(t)\right|$$ (1)

In Equation (1), the term $\vec{X}(t)$ represents a solution at iteration t, while the term ${\vec{X}}^{*}(t)$ denotes the position of the optimal solution or the food source. The “.” in this Equation signifies a form of pairwise multiplication. The term $\vec{X}(t+1)$ represents the updated position of the changed agent. The two vectors, $\vec{A}$ and $\vec{C}$, undergo updates during iterations as $\vec{A}=2\vec{a}.r_{1}-\vec{a}$ and $\vec{C}=2.r_{2}$. The term $\vec{a}$ undergoes a linear change from 2 that represent the maximum value to 0 that represent the minimum value. The values of $r_{1}$ and $r_{2}$ vary randomly within the range of [0, 1]. The term Guided WOA signifies an improved version of the original WOA algorithm [47]. Guided WOA introduces refinements to address the constraints of the initial WOA by adapting the search strategy through the collaboration of multiple agents. In contrast to the original WOA, which mandates agents to move randomly around each other using Equation (1) for global exploration, the adapted Guided WOA algorithm guides agents collaboratively toward the target or optimal solution. Within the framework of the Guided WOA algorithm, the exploration process undergoes improvement as agents are directed to follow three random agents as opposed to just one. This adjustment is made to prevent agents from being overly swayed by a singular leader position. To foster increased exploration, Equation (1) can be replaced with the subsequent expression:


$$\vec{X}(t+1)=\vec{w_{1}}*{\vec{X}}_{rand1}+\vec{\vartheta }*\vec{w_{2}}*\left({\vec{X}}_{rand2}-{\vec{X}}_{rand3}\right)+\left(1-\vec{\vartheta }\right)*\vec{w_{3}}*(\vec{X}-{\vec{X}}_{rand1})$$ (2)

In Equation (2), the three random solutions are denoted as ${\vec{X}}_{rand1}$, ${\vec{X}}_{rand2}$, and ${\vec{X}}_{rand3}$. The value of the term $\vec{w_{1}}$ is adjusted within the range [0, 0.5]. The terms $\vec{w_{2}}$ and $\vec{w_{3}}$ dynamically vary within the interval [0, 1]. To ensure a smooth transition between exploration and exploitation, the term $\vec{\vartheta }$ decreases exponentially, as opposed to linearly, and its calculation is as follows:


$$\vec{\vartheta }=1-{\left(\frac{t}{{Maxi}_{iter}}\right)}^{2}$$ (3)

where, t is the number of iterations and ${Maxi}_{iter}$ is the maximum number of iterations.

#### Particle swarm optimization algorithm.

**Fig 1:**
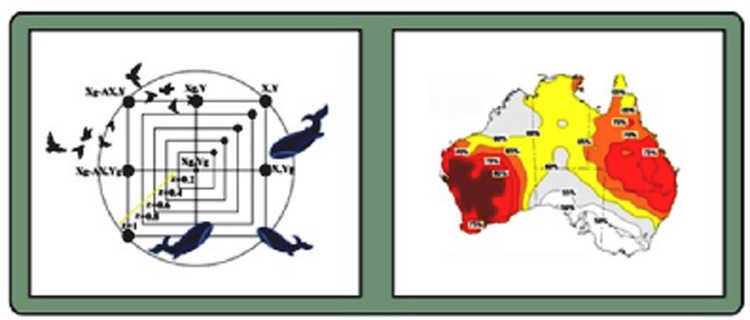
Adaptive dynamic algorithm for Australia rainfall.

In contrast to the WOA algorithm, Particle Swarm Optimization (PSO) mimics the social dynamics observed in swarming patterns of flocks, particularly in birds [48]. In the PSO algorithm, agents actively seek the optimal solution or food by adjusting their positions based on the updated velocity [49]. The approach employs particles (agents), and each agent adheres to specific parameters. The expression $\left(x^{i}\in R^{n}\right)$ denotes a point or location within the search space $R^{n}$. The agents’ positions are determined through a fitness function. The term $\left(v^{i}\right)$ signifies the velocity or rate of change in the positions of the agents. The $\left(p^{i}\right)$ represents the most recent best positions of the particles. Over successive iterations, the positions and velocities of agents are updated. The following equation governs the modification of agents’ positions:


$$x_{\left(t+1\right)}^{i}=x_{t}^{i}+v_{\left(t+1\right)}^{i}$$ (4)

where the position of the new agent position is expressed as $x_{\left(t+1\right)}^{i}$. The velocity of each agent $v_{\left(t+1\right)}^{i}$ is updated as in the following equation:


$$v_{\left(t+1\right)}^{i}=c_{1}r_{1}\left(p_{\left(t\right)}^{i}-x_{\left(t\right)}^{i}\right)+c_{2}r_{2}\left(G-x_{\left(t\right)}^{i}\right)+wv_{\left(t\right)}^{i}$$ (5)

where, in Equation (5), w denotes the inertia weight. The factors $c_{1}$ and $c_{2}$ correspond to cognition and social learning, respectively. The parameter G signifies the global best position, and the values of $r_{1}$ and $r_{2}$ fall within the range [0, 1]. Fig 1 demonstrates the Adaptive dynamic algorithm for Australia rainfall. This figure is composed of two sections, each offering a unique perspective side an adaptive dynamic algorithm and its application to Australian rainfall analysis. The left section portrays an adaptive, nature-inspired algorithm, seemingly modeled after the movement patterns of birds and whales. A central point, labeled as *X*_*g*_ and *Y*_*g*_ are the objective or global target in an optimization framework. The depiction of birds and whales implies a bio-inspired or multi-agent optimization approach, potentially capturing behavioral dynamics or environmental adaptability within the algorithm. The right side represents a geographic representation of Australia and segmented into regions shaded with varying hues. The color gradients represent differential rainfall distributions, with darker shades in red that denotes lower rainfall and lighter shades in yellow that denotes higher and moderate levels. Numerical percentages within each region are correspond to the predicted likelihood for rainfall scenarios.

### The proposed framework

**Fig 2:**
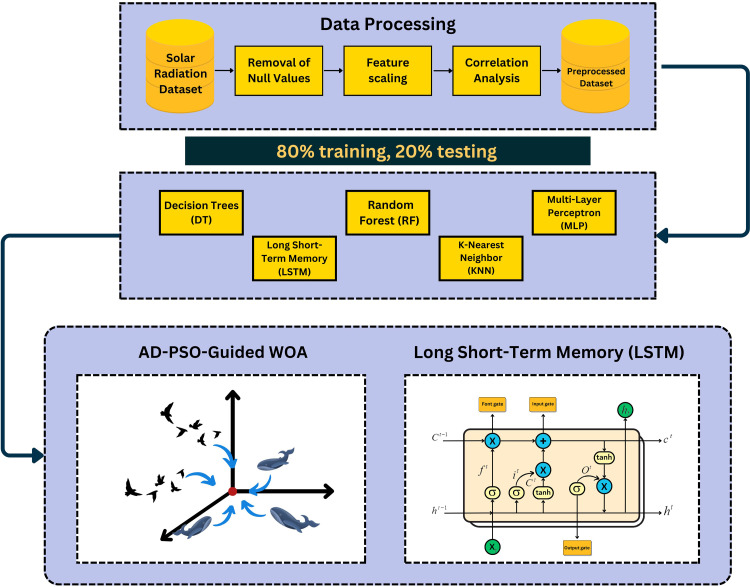
The AD-PSO-Guided WOA LSTM framework.

Data processing, including operations such as data scaling, normalization, and null value removal, is involved in this study. Preparing and improving the input data is the main goal at this point. The study used feature selection algorithms to choose the best features using binary optimization methods to do this. The methods in binary format are Particle Swarm Optimization (PSO), Guided Whale Optimization Algorithm (Guided WOA), Adaptive Dynamic Particle Swarm Guided Whale Optimization Algorithm (AD-PSO-Guided WOA), and Whale Optimization Algorithm (WOA) as shown in Fig 2. The bAD-PSO-Guided WOA algorithm yields the best results for average error, average select size, average fitness, best fitness, worst fitness, and standard deviation fitness. The goal of this stage is to find the best characteristics to enable precise input data prediction. The study proposes the following individual models for the prediction process utilizing the features picked by the bAD-PSO-Guided WOA algorithm: RF, DT, KNN, LSTM, and MLP. The LSTM model produces the most significant outcomes. The LSTM model’s parameters are adjusted via the AD-PSO-Guided WOA, Guided WOA, PSO, and WOA algorithms; the AD-PSO-Guided WOA-LSTM method yields the best outcomes.

#### AD-PSO-guided WOA algorithm.

This section provides a complexity analysis of the AD-PSO-Guided WOA algorithm using Algorithm (1). The effectiveness of an optimizer is gauged by evaluating the fitness function, which is primarily reliant on the regression error rate and the selected features from the input dataset. The optimal solution is determined by a set of features that minimize the feature count and prediction error rate. This study’s solution quality assessment is carried out using the following equation:


$$F_{n}=\alpha Err(O)+\beta \frac{\left|s\right|}{\left|f\right|}$$ (6)

where the error rate of the optimizer is denoted as Err(O), the chosen set of features is represented by s, and f signifies the total number of available features. The value of α is in the range [0, 1] and $\beta =1-h_{1}$. α and β are used for the prediction error rate and the selected feature number. 
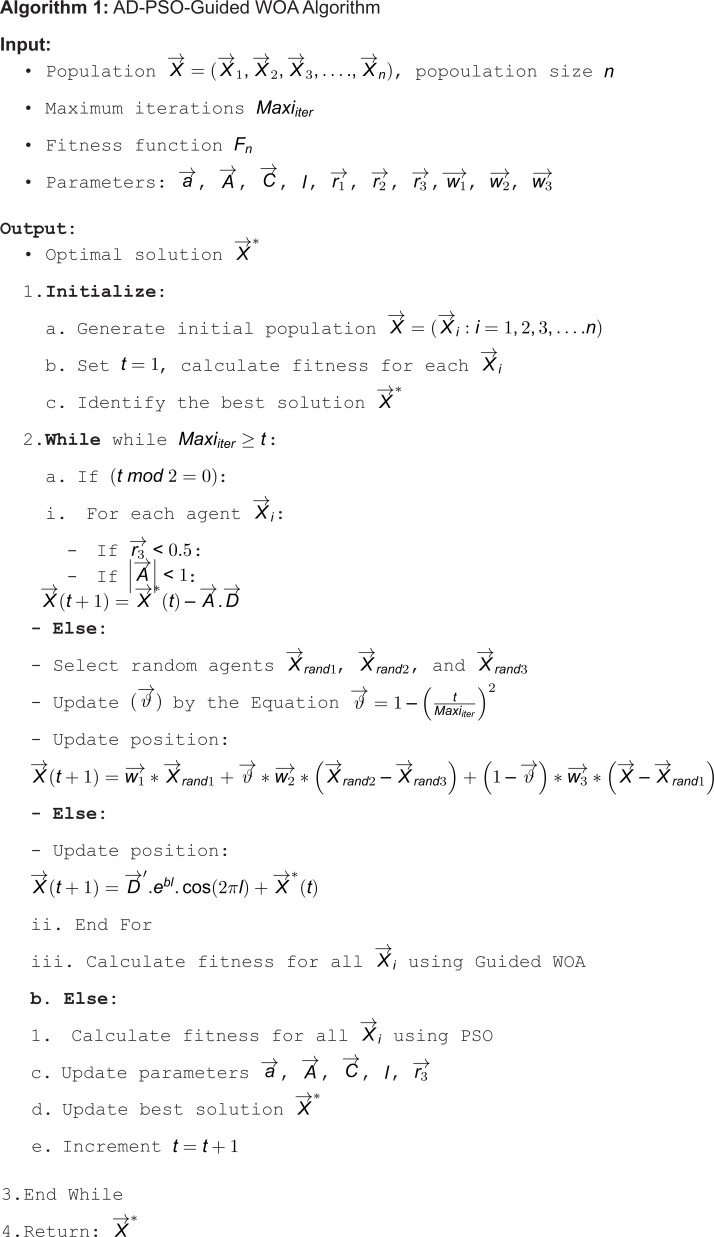


### Evaluation metrics

Table 2 provides an overview of the metrics employed to evaluate the proposed model and the associated mathematical expressions [50]. These metrics include Mean Square Error (MSE), Mean Absolute Error (MAE), Root Mean Square Error (RMSE), Mean Absolute Percentage Error (MAPE), coefficient of determination (R^2^), Normalized RMSE (NRMSE), and Nash–Sutcliffe Efficiency (NSE). In the expressions, *n* denotes the sample size of the dataset and ${Act}_{i}$, ${pre}_{i}$ are the $i^{th}$ and actual and predicted values [51].

**Table 2:** Metrics for evaluating the performance of the proposed model.

Metric	Value
MSE	$\frac{1}{n}\sum_{i=1}^{n}{\left({Act}_{i}-{pre}_{i}\right)}^{2}$
MAE	$\frac{1}{n}\sum_{i=1}^{n}\left|{Act}_{i}-{pre}_{i}\right|$
RMSE	$\sqrt{\frac{1}{n}\sum_{i=1}^{n}{\left({Act}_{i}-{pre}_{i}\right)}^{2}}$
MAPE	$\left(\frac{1}{n}\sum_{i=1}^{n}\left|\frac{{Act}_{i}-{pre}_{i}}{{Act}_{i}}\right|\right)\times 100$
R^2^	$1-\frac{\sum_{i=1}^{n}{\left({Act}_{i}-{pre}_{i}\right)}^{2}}{\sum_{i=1}^{n}{\left(\left(\sum_{i=1}^{n}{Act}_{i}\right)-{Act}_{i}\right)}^{2}}$
NRMSE	$\begin{array}{c} \frac{RMSE}{mean} \end{array}$
NSE	$1-\frac{\sum_{i=1}^{n}{\left({Act}_{i}-{pre}_{i}\right)}^{2}}{\sum_{i=1}^{n}{\left({pre}_{i}-meanof{pre}_{i}\right)}^{2}}$

## Experimental results and discussion

In various experimental conditions, the proposed algorithm underwent assessment. Traditional mathematical functions were utilized in the tests as benchmarks to determine their minimum values within a specified search area. These functions have been commonly employed in prior research to gauge the efficacy of optimization strategies, and numerous optimization methods are available in existing literature. This research conducted a comparative analysis to showcase the superior performance and effectiveness of AD-PSO-Guided WOA algorithm when compared to other recognized optimization algorithms, namely, Guided WOA, PSO, and WOA. These algorithms were selected based on their widespread recognition and practical significance.

### Feature selection results

This study applied five algorithms for the process of feature selection in the binary format, namely, AD-PSO-Guided WOA, Guided WOA, PSO, and WOA. The best results of average error, average select size, average fitness, best fitness, worst fitness, and standard deviation fitness are obtained using the bAD-PSO-Guided WOA algorithm, as shown in Table 3.

**Table 3:** Performance of the bAD-PSO-Guided WOA algorithm compared with another algorithm.

	bAD-PSO-Guided WOA	bGuided WOA	bPSO	bWOA
**Average error**	0.39463	0.41314	0.43573	0.46057
**Average select size**	0.36257	0.56257	0.56257	0.48537
**Average fitness**	0.47297	0.48917	0.48757	0.49527
**Best fitness**	0.37477	0.40947	0.46787	0.47307
**Worst fitness**	0.47327	0.47637	0.53557	0.54927
**Standard deviation fitness**	0.29527	0.29997	0.29937	0.30057

The binary encoding method in the algorithm represents features through binary vectors, where a 1 signifies a selected feature and a 0 denotes an unselected one. The AD-PSO-Guided WOA algorithm refines these vectors through an iterative process, dynamically balancing exploration and exploitation. This optimization is directed by a fitness function that simultaneously evaluates prediction accuracy and the number of features, ensuring an effective and efficient search for the optimal feature subset. A sigmoid-based transfer function was employed to transform continuous values into binary probabilities, facilitating a smooth and continuous transition between values. The feature selection process is governed by a randomly applied threshold, which determines feature inclusion based on these probabilistic outcomes. This approach adheres to established best practices in the domain of binary optimization, ensuring both precision and adaptability. The sigmoid transfer function which computes probabilities is given as:


$$S(v)=\frac{1}{1+e^{v}}$$ (7)

where, v is the position update.

For the process of selection, the algorithm uses a threshold given as:


S(v)>rand() (8)

where, the feature selected is (1) and not selected is (0).

### Prediction results

An additional experiment was carried out to demonstrate the impact of the feature selection on the prediction results. Utilizing characteristics chosen through bAD-PSO-Guided WOA, individual models were applied to predict the input data.

The bAD-PSO-Guided WOA algorithm was employed to enhance the network’s features and optimize performance. The prediction results for various individual models post-feature selection are presented in Table 4. The particular models specified in the table consist of the RF, DT, KNN, LSTM, and MLP. The LSTM model achieved the best results of 0.074, 0.114433, 0.13598, 0.02913, 0.835773, 25.44545, 0.55237, and 1.259669895 for MSE, RMSE, MAE, MAPE, R^2^, NRMSE, NSE and fitted time, respectively. Functioning as a fitness function, the LSTM model is optimized using the AD-PSO-Guided WOA algorithm and additional optimization models, namely, Guided WOA, PSO, and WOA.

**Table 4:** Prediction results of individual models.

Models	MSE	RMSE	MAE	MAPE	R^2^	NRMSE	NSE	Fitted Time
**LSTM**	0.074	0.114433	0.13598	0.02913	0.835773	25.44545	0.55237	1.259669895
**MLP**	0.106	0.298536	0.24854	0.03802	0.748793	56.32771	0.584485	1.282322283
**RF**	0.108	0.301563	0.26056	0.05105	0.758147	56.97876	0.608482	2.570771093
**DT**	0.110	0.305166	0.25578	0.04008	0.759423	57.75364	0.637372	1.437850113
**KNN**	0.112	0.309256	0.26207	0.02761	0.775984	58.63317	0.670596	7.007211561

Table 5 presents the results for the prediction of the optimization algorithms, employing the LSTM model as the fitness function. The outcomes of AD-PSO-Guided WOA in conjunction with LSTM are juxtaposed against those Guided WOA, PSO, and WOA with LSTM, illustrating the superiority of the proposed approach (AD-PSO-Guided WOA-LSTM). The AD-PSO-Guided WOA-LSTM approach exhibited outstanding performance, showcasing MSE, RMSE, MAE, MAPE, R^2^, NRMSE, NSE, and fitted time values of 0.0011, 0.0286, 0.0351, 0.0007, 0.9636, 13.76, 0.9887, and 0.1277, respectively.

**Table 5:** Performance of the proposed AD-PSO-Guided WOA-LSTM algorithm compared with another algorithm.

Models	MSE	RMSE	MAE	MAPE	R^2^	NRMSE	NSE	Fitted Time
**AD-PSO-Guided WOA-LSTM**	0.0011	0.0286	0.0351	0.0007	0.9636	13.76	0.9887	0.1277
**Guided WOA-LSTM**	0.0057	0.0437	0.0521	0.0035	0.9547	16.69	0.8859	0.1318
**PSO-LSTM**	0.0089	0.0658	0.0855	0.0061	0.9455	18.71	0.8164	0.1323
**WOA-LSTM**	0.0112	0.0979	0.0953	0.0088	0.9036	20.99	0.7354	0.1329

Table 6 illustrates the descriptive analysis of the results obtained through the optimized LSTM utilizing various optimization algorithms. The table compares the performance of LSTM models optimized with different algorithms: AD-PSO-Guided WOA-LSTM, Guided WOA-LSTM, PSO-LSTM, and WOA-LSTM. The AD-PSO-Guided WOA-LSTM demonstrates lower outcomes across the key metrics, underscoring its superior efficiency and predictive precision relative to the other models. WOA-LSTM stands out with the highest recorded metrics, making it the least stable among the evaluated models.

**Table 6:** Descriptive analysis of the outcomes by the optimized LSTM using several optimization algorithms.

	AD-PSO-Guided WOA-LSTM	Guided WOA-LSTM	PSO-LSTM	WOA-LSTM
**Number of values**	5	5	5	5
**Minimum**	0.02769	0.04271	0.06586	0.0858
**25% Percentile**	0.02819	0.04321	0.06586	0.08839
**Median**	0.02869	0.04371	0.06586	0.09798
**75% Percentile**	0.02883	0.04471	0.07872	0.09898
**Maximum**	0.02897	0.04571	0.08159	0.09998
**Range**	0.001275	0.003	0.01573	0.01418
**Mean**	0.02855	0.04391	0.071	0.09454
**Std. Deviation**	0.0004927	0.001095	0.007331	0.005964
**Std. Error** **of Mean**	0.0002203	0.0004899	0.003279	0.002667
**Sum**	0.1427	0.2196	0.355	0.4727

Table 7 displays the ANOVA results for the proposed AD-PSO-Guided WOA-LSTM algorithm in the context of rainfall prediction. The effectiveness of the proposed AD-PSO-Guided WOA-LSTM approach in optimizing the objective function is validated through a comparative analysis with alternative optimization algorithms employing the LSTM model.

**Table 7:** The outcomes of the ANOVA results for the proposed AD-PSO-Guided WOA-LSTM algorithm for rainfall prediction.

ANOVA table	SS	DF	MS	F (DFn, DFd)	P value
**Treatment (between columns)**	0.01281	3	0.004269	F (3, 16) = 188.1	P<0.0001
**Residual (within columns)**	0.00036	16	0.000022		
**Total**	0.01317	19			

**Fig 3:**
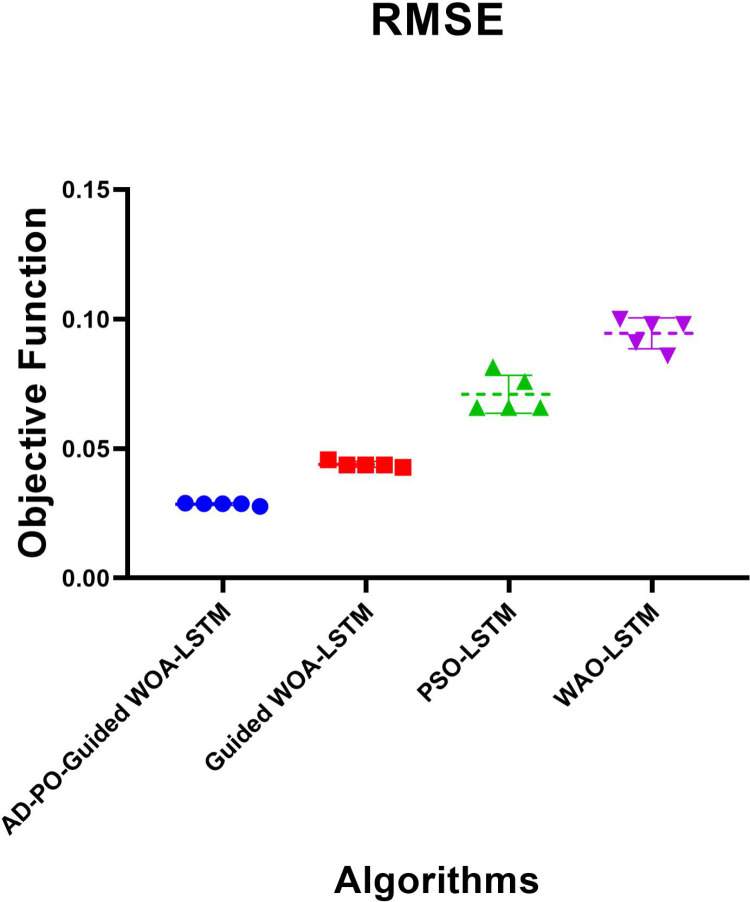
The AD-PSO-Guided WOA LSTM algorithm RMSE is based on the objective function compared to different algorithms.

RMSE results obtained from the proposed AD-PSO-Guided WOA-LSTM algorithm and other algorithms are depicted in the RMSE plots shown in Fig 3. The RMSE plot illustrates that the proposed approach outperformed the different optimization algorithms using the LSTM model.

**Fig 4:**
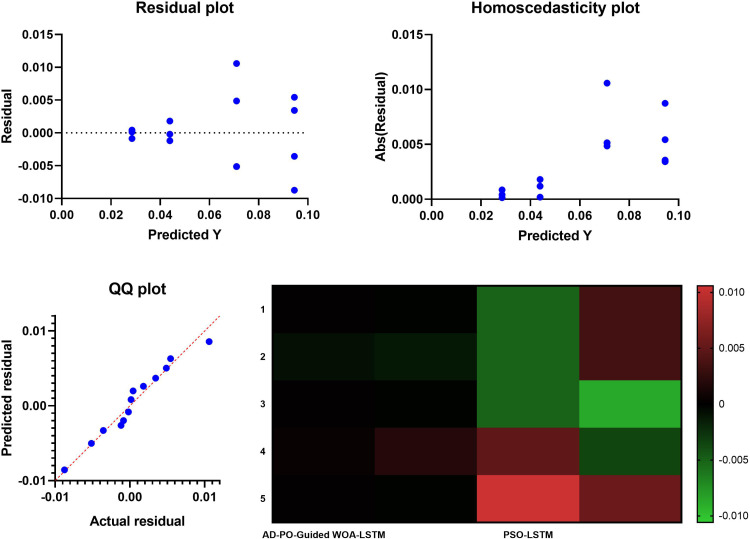
Analysis plots of the obtained results using the proposed AD-PSO-Guided WOA LSTM algorithm.

Fig 4 presents a collection of plots, including the residual plot, QQ plot, homoscedasticity plot, and heatmap. These visualizations utilize residual plots, quartile-quartile (QQ) plots, and homoscedasticity to highlight the efficiency and robustness of the proposed AD-PSO-Guided WOA-LSTM algorithm. The QQ plot, with values that closely adhere to a linear trend, provides evidence for the selected features’ efficacy in categorical classification. The information depicted in the residual and homoscedasticity plots further supports these findings. Additionally, Fig 4 heatmap underscores the superiority of the proposed AD-PSO-Guided WOA-LSTM algorithm, clearly indicating its outperformance compared to other algorithms. The heatmap serves as additional confirmation of the effectiveness of the AD-PSO-Guided WOA-LSTM algorithm in achieving optimal results compared to alternative feature selection strategies. The analytical plots presented in Fig 4 collectively affirm the success of the AD-PSO-Guided WOA-LSTM algorithm in addressing optimization concerns within the context of rainfall prediction.

Table 8 demonstrates the hyperparameter settings for the optimization algorithms used in this study.

**Table 8:** Hyperparameter settings for the optimization algorithms.

Algorithm	Hyperparameters	Value
**AD-PSO-Guided WOA**	Population size (n)	50
	Maximum iterations (T)	100
	Exploration/ Exploitation split	50/50
	Decreasing parameter (a)	2
	Sigmoid transfer threshold	0.5
**PSO**	Inertia weight (ω)	0.8
	Cognitive coefficient $\left(c_{1}\right)$	2
	Social coefficient $\left(c_{2}\right)$	2
	Velocity clamping factor	0.4
**Guided WOA**	Search parameter (a)	2
	Random movement parameter (P)	0.5
	Number of guiding agents	3
**WOA**	Control parameter (b)	1
	Random parameter (r)	0.3

The total computational cost for the proposed AD-PSO-Guided WOA-LSTM algorithm, Guided WOA-LSTM algorithm, PSO-LSTM algorithm, and WOA-LSTM algorithm is demonstrated in Table 9. The total computational cost $\left(T_{total}\right)$ is computed as:

**Table 9:** Total computational cost of the proposed AD-PSO-Guided WOA-LSTM algorithm compared with another algorithm.

Models	Total computational cost
**AD-PSO-Guided WOA-LSTM**	112.7
**Guided WOA-LSTM**	113.2
**PSO-LSTM**	113.6
**WOA-LSTM**	114.3


$$T_{total}=O(n.t.f)$$ (9)

where, n represents the population size, t represents the number of iterations, and f represents the fitness evaluations per iteration.

As demonstrated in Table 9, the best result for the total computational cost is obtained by AD-PSO-Guided WOA-LSTM algorithm. The total computational cost for AD-PSO-Guided WOA-LSTM algorithm is 112.7, which represents the most computationally efficient approach, balancing exploration and exploitation. The worth results is obtained by WOA-LSTM algorithm with total computational cost of 114.3.

## Conclusion and future work

Forecasting rainfall involves examining and categorizing various types of rainfall based on factors like intensity, duration, distribution, and associated meteorological conditions. Understanding rainfall patterns and prediction plays a crucial role in diverse applications such as agriculture, water resource management, weather forecasting, and climate studies. Analyzing different facets of rainfall makes it possible to make well-informed decisions in areas like agricultural planning, efficient water resource utilization, accurate weather predictions, and gaining deeper insights into climate-related phenomena. To tackle the challenges associated with rainfall prediction, the paper introduces the Adaptive Dynamic Particle Swarm optimization augmented with the Guided Whale Optimization Algorithm. This hybrid algorithm is applied explicitly for rainfall prediction. The binary format of AD-PSO-Guided WOA functions as a feature selection algorithm, aiding in identifying the most critical features within the dataset. Subsequently, the selected features are utilized to train five individual models: RF, DT, KNN, LSTM, and MLP. Among the individual models, LSTM emerges as the top performer. Further optimization of the LSTM model’s hyperparameters is achieved using the AD-PSO-Guided WOA algorithm. The outcomes affirm the superior performance and effectiveness of the proposed approach (AD-PSO-Guided WOA-LSTM) compared to alternative optimization methods, demonstrating an R^2^ value of 0.9636. This suggests that the proposed algorithmic combination offers promising results for enhancing the accuracy of rainfall prediction models. There are numerous possibilities for advancing and expanding the current study. Here are potential areas for future investigation, such as delving into the inclusion of extra pertinent data sources like satellite imagery, soil moisture data, or geographical information. The integration of diverse datasets has the potential to offer a more comprehensive insight into the elements impacting rainfall. Extending the study’s temporal range by considering lengthier time intervals or integrating real-time data for ongoing monitoring is another avenue to explore. Furthermore, examining the model’s adaptability across diverse geographical locations can provide valuable insights into its resilience and generalization capabilities. We recognize that the proposed algorithm presents some shortcomings, particularly in addressing computational demands, enhancing dataset diversity, and mitigating dependence on manual hyperparameter adjustments. Future efforts will focus on integrating parallel computing frameworks to alleviate computational overhead, conducting extensive evaluations across heterogeneous datasets to validate generalizability, and leveraging advanced explainable AI methodologies to unravel complex feature interdependencies. Moreover, the adoption of automated hyperparameter optimization strategies, such as Bayesian techniques or evolutionary algorithms, could further streamline the tuning process and enhance predictive robustness. These avenues of improvement hold promise for augmenting the scalability, adaptability, and interpretability of the algorithm in diverse real-world applications.

Acknowledgments 

Princess Nourah bint Abdulrahman University Researchers Supporting Project number (PNURSP2025R308), Princess Nourah bint Abdulrahman University, Riyadh, Saudi Arabia.

## Supporting Information

S1 Appendix Applied the proposed model on a another public dataset. (DOCX)

## References

[1] Parmar A, Mistree K, Sompura M. Machine learning techniques for rainfall prediction: A review. International conference on innovations in information embedded and communication systems (Vol 3). 2017. doi: DOIoridentifierneeded

[2] Abhishek K, Kumar A, Ranjan R, Kumar S. A rainfall prediction model using artificial neural network. 2012 IEEE Control and System Graduate Research Colloquium. IEEE; 2012. p. 82–7.

[3] Nayak D, Mahapatra A, Mishra P. A survey on rainfall prediction using artificial neural network. Int. J. Comput. Appl. 2013;72(16):32–40.

[4] Lee S, Cho S, Wong P. Rainfall prediction using artificial neural networks. J. Geogr. Inf. Decis. Anal. 1998;2(2):233–42.

[5] Rahman A-U, Abbas S, Gollapalli M, Ahmed R, Aftab S, Ahmad M, et al. Rainfall prediction system using machine learning fusion for smart cities. Sensors (Basel). 2022;22(9):3504. doi: 10.3390/s22093504 35591194 PMC9099780

[6] Kashiwao T, Nakayama K, Ando S, Ikeda K, Lee M, Bahadori A. A neural network-based local rainfall prediction system using meteorological data on the Internet: A case study using data from the Japan Meteorological Agency. Appl. Soft Comput. 2017;56:317–30.

[7] Shams M, El-Kenawy E, Ibrahim A, Elshewey A. A hybrid dipper throated optimization algorithm and particle swarm optimization (DTPSO) model for hepatocellular carcinoma (HCC) prediction. Biomed. Signal Process. Control. 2023;85:104908.

[8] Elshewey AM, Shams MY, Tawfeek SM, Alharbi AH, Ibrahim A, Abdelhamid AA, et al. Optimizing HCV disease prediction in Egypt: The hyOPTGB framework. Diagnostics. 2023;13(22):3439. 37998575 10.3390/diagnostics13223439 PMC10670002

[9] Qiu M, Zhao P, Zhang K, Huang J, Shi X, Wang X, et al. A short-term rainfall prediction model using multi-task convolutional neural networks. In: 2017 IEEE international conference on data mining (ICDM). 2017. p. 395–404.

[10] El-Kenawy ES, Abdelhamid AA, Alrowais F, Abotaleb M, Ibrahim A, Khafaga DS. Al-Biruni Based Optimization of Rainfall Forecasting in Ethiopia. Computer Systems Science & Engineering. 2023;46(1).

[11] Liu Q, Zou Y, Liu X, Linge N. A survey on rainfall forecasting using artificial neural network. Int. J. Embed. Syst. 2019;11(2):240–9.

[12] Mengistu W, Workie A, Mohammed SA. Physical and cup quality attributes of arabica coffee (Coffea arabica L.) varieties grown in highlands of Amhara Region, Northwestern Ethiopia. Int. J. Agron. 2020;2020(1):6420363. doi: 10.1155/2020/6420363

[13] Mishra N, Soni H, Sharma S, Upadhyay A. Development and analysis of artificial neural network models for rainfall prediction by using time-series data. Int. J. Intell. Syst. Appl. 2018;10(1):16. doi: PleaseprovidetheDOIorotheridentifiers

[14] Endalie D, Tegegne T. Designing a hybrid dimension reduction for improving the performance of Amharic news document classification. PLoS One. 2021;16(5):e0251902. doi: 10.1371/journal.pone.0251902 34019571 PMC8139506

[15] El-Shafie A, Mukhlisin M, Najah A, Taha M. Performance of artificial neural network and regression techniques for rainfall-runoff prediction. Int. J. Phys. Sci. 2011;6(8):1997–2003.

[16] Hasan N, Nath N, Rasel R. A support vector regression model for forecasting rainfall. In: 2015 2nd International Conference on Electrical Information and Communication Technologies (EICT). 2015. p. 554–9.

[17] He X, Guan H, Zhang X, Simmons C. A wavelet-based multiple linear regression model for forecasting monthly rainfall. Int. J. Climatol. 2014;34(6).

[18] Ramana GV. Regression analysis of rainfall and runoff process of a typical watershed. Int. J. 2014;3(1):16–26.

[19] Hong W-C, Pai P-F. Potential assessment of the support vector regression technique in rainfall forecasting. Water Resour Manage. 2006;21(2):495–513. doi: 10.1007/s11269-006-9026-2

[20] Goyal M. Monthly rainfall prediction using wavelet regression and neural network: an analysis of 1901–2002 data, Assam, India. Theor. Appl. Climatol. 2014;118:25–34.

[21] Danandeh Mehr A, Nourani V, Karimi Khosrowshahi V, Ghorbani M. A hybrid support vector regression–firefly model for monthly rainfall forecasting. Int. J. Environ. Sci. Technol. 2019;16:335–46.

[22] Pai P, Hong W. A recurrent support vector regression model in rainfall forecasting. Hydrol. Process. 2007;21(6):819–27.

[23] Hossain I, Esha R, Alam Imteaz M. An attempt to use non-linear regression modelling technique in long-term seasonal rainfall forecasting for australian capital territory. Geosciences. 2018;8(8):282.

[24] Chandniha S, Kansal M. Rainfall estimation using multiple linear regression based statistical downscaling for Piperiya watershed in Chhattisgarh. J. Agrometeorology. 2016;18(1):106–12.

[25] Kaggle dataset. Rain in Australia. Available at https://www.kaggle.com/code/ahmedraft/rain-in-australia/input

[26] Alkhammash E, Hadjouni M, Elshewey A. A hybrid ensemble stacking model for gender voice recognition approach. Electronics. 2022;11(11):1750.

[27] Shams M, Tarek Z, Elshewey A, Hany M, Darwish A, Hassanien A. A machine learning-based model for predicting temperature under the effects of climate change. In: The Power of Data: Driving Climate Change with Data Science and Artificial Intelligence Innovations. 2023. p. 61–81.

[28] Elshewey A, Shams M, Elhady A, Shohieb S, Abdelhamid A, Ibrahim A, et al. A novel WD-SARIMAX model for temperature forecasting using daily delhi climate dataset. Sustainability. 2022;15(1):757.

[29] Alkhammash EH, Assiri SA, Nemenqani DM, Althaqafi RMM, Hadjouni M, Saeed F, et al. Application of machine learning to predict COVID-19 Spread via an Optimized BPSO Model. Biomimetics (Basel). 2023;8(6):457. doi: 10.3390/biomimetics8060457 37887588 PMC10604133

[30] Xue H, Huynh D, Reynolds M. SS-LSTM: A hierarchical LSTM model for pedestrian trajectory prediction. In: 2018 IEEE Winter Conference on Applications of Computer Vision (WACV). IEEE; 2018. p. 1186–94.

[31] Shams M, Tarek Z, El-kenawy E, Eid M, Elshewey A. Predicting Gross Domestic Product (GDP) using a PC-LSTM-RNN model in urban profiling areas. Comput. Urban Sci. 2024;4(1):3.

[32] Elshewey AM, Shams MY, Tarek Z, Megahed M, El-Kenawy ES, El-dosuky MA. Weight prediction using the hybrid stacked-lstm food selection model. Comput Syst Sci Eng. 2023;46(1):765–81.

[33] Sonawane J, Patil D. Prediction of heart disease using multilayer perceptron neural network. In: Proceedings of the International Conference on Information Communication and Embedded Systems (ICICES2014). IEEE; 2014. p. 1–6.

[34] Popescu M, Balas V, Perescu-Popescu L, Mastorakis N. Multilayer perceptron and neural networks. WSEAS Trans. Circuits Sys. 2009;8(7):579–88.

[35] Curteanu S, Cartwright H. Neural networks applied in chemistry. I. Determination of the optimal topology of multilayer perceptron neural networks. J. Chemom. 2011;25(10):527–49.

[36] Ortiz-Bejar J, Graff M, Tellez E, Jacobo J. K-nearest neighbor regressors optimized by using random search. In: 2018 IEEE International Autumn Meeting on Power, Electronics and Computing (ROPEC). IEEE; 2018. p. 1–5.

[37] Imandoust S, Bolandraftar M. Application of k-nearest neighbor (knn) approach for predicting economic events: Theoretical background. Int. J. Eng. Res. Appl. 2013;3(5):605–10.

[38] Priyam A, Abhijeeta GR, Rathee A, Srivastava S. Comparative analysis of decision tree classification algorithms. Int. J. Curr. Eng. Technol. 2013;3(2):334-7.

[39] Patel H, Prajapati P. Study and analysis of decision tree based classification algorithms. Int. J. Comput. Sci. Eng. 2018;6(10):74–8.

[40] Li R, Belford G. Instability of decision tree classification algorithms. In: Proceedings of the eighth ACM SIGKDD international conference on Knowledge discovery and data mining. 2002. p. 570–5.

[41] Graw J, Wood W, Phrampus B. Predicting global marine sediment density using the random forest regressor machine learning algorithm. J Geophys. Res. Solid Earth. 2021;126(1):e2020JB020135.

[42] El Mrabet Z, Sugunaraj N, Ranganathan P, Abhyankar S. Random forest regressor-based approach for detecting fault location and duration in power systems. Sensors (Basel). 2022;22(2):458. doi: 10.3390/s22020458 35062419 PMC8779374

[43] Rabehi A, Kumar P. Improving tuberculosis diagnosis and forecasting through machine learning techniques: a systematic review. Metaheuristic Optimization Review. 2024;1(1):35-44.

[44] Gaber KS, Elsebaey MA, Ibrahim AA. Weather prediction: predicting rain using weather conditions. J. Artif. Intell. Metaheuristics. 2024;8(1):60–9.

[45] Ghoneim S, Farrag T, Rashed A, El-Kenawy E, Ibrahim A. Adaptive dynamic meta-heuristics for feature selection and classification in diagnostic accuracy of transformer faults. IEEE Access. 2021;9:78324–40.

[46] Mirjalili S, Mirjalili S, Saremi S, Mirjalili S. Whale optimization algorithm: theory, literature review, and application in designing photonic crystal filters. Nature-inspired optimizers: theories, literature reviews and applications. 2020. p. 219–38.

[47] Nazir M, Alturise F, Alshmrany S, Nazir H, Bilal M, Abdalla A, et al. Wind generation forecasting methods and proliferation of artificial neural network: A review of five years research trend. Sustainability. 2020;12(9):3778.

[48] El-kenawy E, Khodadadi N, Mirjalili S, Abdelhamid A, Eid M, Ibrahim A. Greylag goose optimization: Nature-inspired optimization algorithm. Expert Syst Appl. 2024;238:122147.

[49] Ibrahim A, Mirjalili S, El-Said M, Ghoneim S, Al-Harthi M, Ibrahim T, et al. Wind speed ensemble forecasting based on deep learning using adaptive dynamic optimization algorithm. IEEE Access. 2021;9:125787–804.

[50] Tarek Z, Shams MY, Towfek SK, Alkahtani HK, Ibrahim A, Abdelhamid AA, et al. An optimized model based on deep learning and gated recurrent unit for COVID-19 death prediction. Biomimetics (Basel). 2023;8(7):552. doi: 10.3390/biomimetics8070552 37999193 PMC10669113

[51] A Deep learning prediction model to predict sustainable development in Saudi Arabia. Appl Math Inf Sci. 2024;18(6):1345–66. doi: 10.18576/amis/180615

