# Supplementary material for: An enhanced adaptive dynamic metaheuristic optimization algorithm for rainfall prediction depends on long short-term memory
Source: PLoS One. 2025 Jun 2;20(6):e0317554. doi: 10.1371/journal.pone.0317554 (PMC12129181; doi:10.1371/journal.pone.0317554)
Supplement: S1 Appendix — (DOCX) [file pone.0317554.s001.docx]

**S1 Appendix**

To test the performance of the proposed model, we applied the proposed model on a public dataset available at <https://www.kaggle.com/datasets/shiratorizawa/ncarcsv2> and consists of 622392 instances. The dataset features are latitude, longitude, dates, mean sea level pressure, U-wind, V-wind, temperature, vertical wind velocity, relative humidity, precipitable water, and rainfall. The prediction results for various individual models post-feature selection using the binary format of AD-PSO-Guided WOA model are presented in S1 Table . The particular models specified in the table consist of the RF, DT, KNN, LSTM, and MLP. The LSTM model achieved the best results of 0.058, 0.0991, 0.1206, 0.0138, 0.8204, 31.430, 0.5370, and 2.3443 for MSE, RMSE, MAE, MAPE, R^2^, NRMSE, NSE and fitted time, respectively. Functioning as a fitness function, the LSTM model is optimized using the AD-PSO-Guided WOA algorithm and additional optimization models, namely, Guided WOA, PSO, and WOA. S2 Table presents the results for the prediction of the optimization algorithms, employing the LSTM model as the fitness function. The outcomes of AD-PSO-Guided WOA in conjunction with LSTM are juxtaposed against those Guided WOA, PSO, and WOA with LSTM, illustrating the superiority of the proposed approach (AD-PSO-Guided WOA-LSTM). The AD-PSO-Guided WOA-LSTM approach exhibited outstanding performance, showcasing MSE, RMSE, MAE, MAPE, R^2^, NRMSE, NSE, and fitted time values of 0.0275, 0.0629, 0.0707, 0.0268, 0.9879, 14.89, 1.034, and 1.153, respectively.

**S1 Table .** Prediction results of individual models.

| **Models** | **MSE** | **RMSE** | **MAE** | **MAPE** | **R^2^** | **NRMSE** | **NSE** | **Fitted Time** |
| --- | --- | --- | --- | --- | --- | --- | --- | --- |
| **LSTM** | 0.058 | 0.0991 | 0.1206 | 0.0138 | 0.8204 | 31.430 | 0.5370 | 2.3443 |
| **MLP** | 0.090 | 0.2832 | 0.2332 | 0.0227 | 0.7334 | 56.312 | 0.5691 | 2.4170 |
| **RF** | 0.096 | 0.3162 | 0.2452 | 0.0357 | 0.7628 | 56.963 | 0.5931 | 3.1254 |
| **DT** | 0.105 | 0.3472 | 0.2646 | 0.0413 | 0.8251 | 57.438 | 0.6220 | 3.8169 |
| **KNN** | 0.174 | 0.4875 | 0.3482 | 0.0691 | 0.9524 | 59.715 | 0.7451 | 5.2259 |

**S2 Table .** Performance of the proposed AD-PSO-Guided WOA-LSTM algorithm compared with another algorithm.

| **Models** | **MSE** | **RMSE** | **MAE** | **MAPE** | **R^2^** | **NRMSE** | **NSE** | **Fitted Time** |
| --- | --- | --- | --- | --- | --- | --- | --- | --- |
| **AD-PSO-Guided WOA-LSTM** | 0.0275 | 0.0629 | 0.0707 | 0.0268 | 0.9879 | 14.89 | 1.034 | 1.153 |
| **Guided WOA-LSTM** | 0.0373 | 0.0689 | 0.0785 | 0.0298 | 0.9873 | 17.71 | 0.925 | 1.575 |
| **PSO-LSTM** | 0.0452 | 0.0931 | 0.1308 | 0.0414 | 1.0571 | 19.84 | 0.989 | 2.047 |
| **WOA-LSTM** | 0.0665 | 0.0984 | 0.1583 | 0.0752 | 1.4845 | 22.01 | 1.374 | 3.158 |
